# Anti-osteoporotic Drug Utilization Rates for Secondary Prevention Among Patients with Osteoporotic Fractures

**DOI:** 10.5041/RMMJ.10473

**Published:** 2022-07-31

**Authors:** Cenk Aypak, Mustafa A. Bircan, Ayşe Özdemir

**Affiliations:** Department of Family Medicine, University of Health Sciences, Ankara Dışkapı Yıldırım Beyazıt Training and Research Hospital, Ankara, Turkey

**Keywords:** Anti-osteoporotic drugs, drug utilization, fracture, osteoporosis, secondary prevention

## Abstract

**Objectives:**

Anti-osteoporotic drugs (AOD) are essential for secondary prevention of osteoporotic fracture (OF) in patients with established osteoporosis. However, data about AOD utilization rates are scarce among patients with OF. This study was therefore aimed at determining the AOD utilization rates among those particularly vulnerable patients.

**Materials and Methods:**

This cross-sectional study followed the medical records of patients with OF starting from their first OF diagnosis date. Each patient’s preventive osteoporosis treatments (vitamin D, calcium+vitamin D) and AOD utilization rate were recorded for a 12-month period following OF diagnosis.

**Results:**

A total of 210 patients (168 females, mean age: 67.8±11.9 years; 42 males, mean age 62.4±16.1 years) were enrolled in the study. Of these, 65.7% (*n*=138) did not use any medication for primary protection against osteoporosis before OF diagnosis. The ratio of patients not using any type of medication for secondary prevention after OF increased from 26.5% to 51% during a 12-month period. In addition, by one year following diagnosis, AOD usage rate had decreased from 62.3% to 41.3%.

**Conclusion:**

The AOD usage rates for secondary prevention of OF were insufficient, and cessation rates were high. Identification of factors associated with decreased AOD utility rates will provide important information for guiding patient follow-up in order to reduce the occurrence of OF.

## INTRODUCTION

Osteoporosis is a disease characterized by bone mass reduction and deterioration of bone architecture, leading to impaired skeletal strength and an increased predisposition for fractures.^1^ It is a worldwide health problem particularly in the aging population and is often underdiagnosed and undertreated.^2^ The most significant osteoporosis complication is osteoporotic fracture (OF). The lifetime risk of OF incidence is higher in women than in men: the 10-year fracture risk at 50 years of age is 9.8% in women and 7.1% in men, which increases to 21.7% and 8%, respectively, by 80 years of age.^3^ One in three postmenopausal women and one in five men over 50 years of age will develop fractures with no significant trauma, associated with a significant increase in morbidity and mortality, and a high economic burden.^4^ Osteoporotic fractures are prevalent among the elderly, and all major ones are associated with increased mortality risk.^5^,^6^ Therefore, the main treatment goal includes reducing bone loss, increasing bone mass, protecting and improving the bone architecture, minimizing falls, and reducing OF risk. Several pharmacologic therapies have proven efficacy for reducing OF risk, including bisphosphonates, raloxifene, teriparatide, denosumab, calcitonin, and abaloparatide.^7^,^8^ Bisphosphonates are the most widely used drugs for the prevention and treatment of osteoporosis.^9^

The costs and implications of OF on national health care systems are increasing rapidly due to the high incidence of OF. Hence, intense efforts are being made to prevent a second OF in people who have already experienced a first one.^10^

Previous fractures are one of the significant risk factors for subsequent OF.^11^ Therefore, encouraging patients to be persistent and compliant with their anti-osteoporosis treatments is essential for preventing OF. Previous studies have shown that patients who are persistent and compliant to their anti-osteoporosis treatments have a lower percentage of OF incidence.^6^,^11^ However, prolonged treatment durations may predispose patients to abandon their treatments. Indeed, poor adherence and lack of persistence with treatment are common among patients who are prescribed with anti-osteoporotic drugs (AOD).^12^,^13^ Moreover, in many countries, the post-fracture care gap is a problem, as patients with hip or other fractures are often not prescribed osteoporosis therapy.^12^–^17^ In Turkey, there is no generally accepted treatment and follow-up program other than surgical intervention following OF. In addition, data are lacking regarding medical treatment and patient compliance following OF. Hence, this study was aimed at evaluating the use of AOD in OF patients.

## METHODS

This cross-sectional study was designed to evaluate AOD usage among the patients with OF. Over a two-year period, 214 patients with OF were admitted to a tertiary trauma hospital in Ankara, Turkey. Patient diagnoses were recorded in the hospital’s medical software according to the following International Classification of Diseases (ICD-10) codes: M80.0, Postmenopausal osteoporosis with pathological fracture; M80.1, Postoophorectomy osteoporosis with pathological fracture; M80.2, Osteoporosis of disuse with pathological fracture; M80.3, Postsurgical malabsorption osteoporosis with pathological fracture; M80.4, Drug-induced osteoporosis with pathological fracture; M80.5, Idiopathic osteoporosis with pathological fracture; M80.8, Other osteoporosis with pathological fracture; and M80.9, Unspecified osteoporosis with pathological fracture.

Usage of preventive osteoporosis treatments (vitamin D, calcium+vitamin D) and AOD (bisphosphonate, calcitonin, raloxifene, teriparatide, selective estrogen receptor modulators [SERMs], strontium renalate, denosumab) had been recorded. Following their initial diagnosis, each patient’s drug utilization processes were recorded in the hospital’s medical software for 12 months from the OF diagnosis date. Drug utilization was followed up every 3 months. Due to failure to obtain drug utilization records after OF, 4 patients were excluded from the study. Another 6 patients were included only in the pre-OF analyses, but were then excluded due to death within 3 months of OF. Descriptive statistical methods (frequency, percentage, mean, standard deviation) were utilized to analyze study data.

This research was approved by the hospital’s institutional review board (no. 23/07; 25 May 2015).

## RESULTS

Of the 210 patients included in the study, 80% were female (*n*=168) and 20% were male (*n*=42); mean age was 67.8±11.9 (range, 25–91 years) and 62.4± 16.1 (range, 21–91 years), respectively. A total of 133 (63.3%) patients were more than 65 years of age.

It was found that 65.7% (*n*=138) of the patients did not use any medication for osteoporosis before OF diagnosis. Although 34.3% (*n*=72) of the patients used medications before their diagnosis, 15.3% (*n*=11) of these patients did not use any AOD.

Of the patients receiving AOD before OF, 95.2% (*n*=58) were taking bisphosphonates, 3.2% (*n*=2) were taking strontium ranelate, and 1.6% (*n*=1) was taking SERM. Among the female patients, 29.2% (*n*=49) were taking AOD prior to OF diagnosis while 70.8% (*n*=119) were not. Following OF, 38.7% (*n*=65) of female patients began to use AOD. However, 32.1% (*n*=54) of the females did not use AOD before or after OF diagnosis. Rates of drug utilization in males before and after OF diagnosis (28.6% and 38.1%, respectively) were similar to those in females.

**Figure 1:**
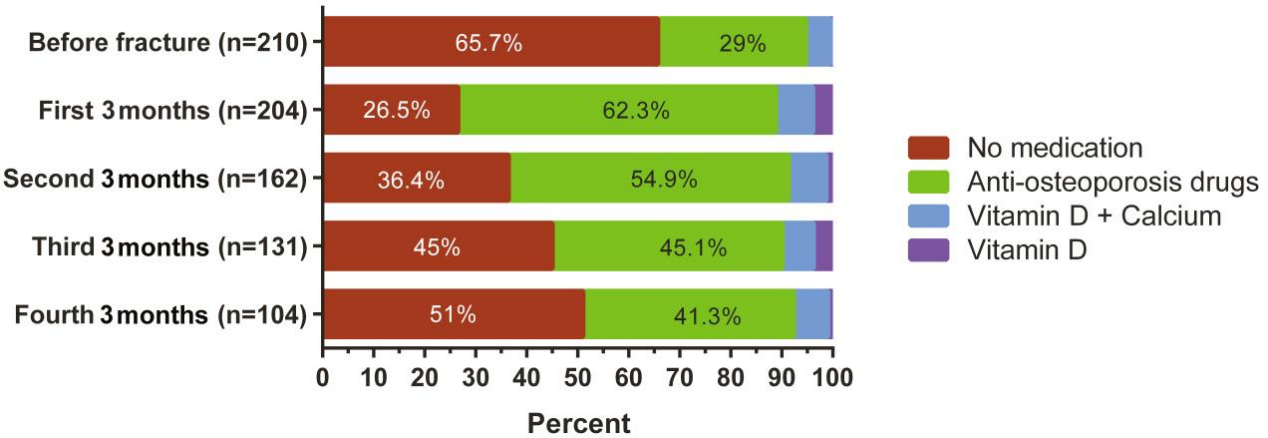
Drug Usage Rates before Fracture and in Succession of 3-Month Periods.

Drug utilization was evaluated every 3 months. During the first 3 months after OF diagnosis, 26.5% (*n*=54) of patients used no osteoporosis medications of any kind. Of those receiving medications, 11.2% (*n*=23) received only vitamin D or calcium+vitamin D, but no AOD medications. One year after OF diagnosis, the AOD usage rate had decreased from 62.3% to 41.3%. The ratio of patients not using osteoporosis medication after fracture increased from 26.5% to 51% over a one-year period. Drug usage rates before and after OF for each three-month period are shown in Figure 1.

Follow-up of patients receiving AOD after osteoporosis diagnosis revealed an AOD usage of 79.4% at the end of month 3; this usage rate decreased to 57.1% over a one-year period. The rate of patients not using any medication increased with each progressive three-month interval.

## DISCUSSION

Osteoporosis is a skeletal disease that decreases bone mineral density and deteriorates bone architecture, thereby significantly increasing the fracture risk.^1^ The most common form of osteoporosis is idiopathic.^18^ In addition, osteoporosis may develop due to secondary causes and several diseases (Cushing’s syndrome, hyperthyroidism, inflammatory bowel disease, rheumatoid arthritis, systemic lupus erythematosus, etc.), and their related drug therapies (glucocorticoids, thyroid management therapy, antidepressants, anticonvulsants, anticoagulants, diuretics, etc.) can cause secondary osteoporosis.^18^ Regardless of the cause of osteoporosis, occurrence of OF is also a risk factor for subsequent fractures.^11^ Hence, administration of AOD is as important as calcium and vitamin D supplementation for prevention of further fractures.

The National Osteoporosis Foundation (NOF) recommends that all osteoporosis patients ensure adequate intake of calcium and vitamin D in their daily diet, whether or not they have experienced a fracture.^19^ They also recommend the use of calcium and vitamin D supplements if a patient’s dietary intake is insufficient. However, taking medications that increase gastric pH, such as proton pump inhibitors or antiacids, and physical conditions such as atrophic gastritis could impair calcium absorption.^20^ Absorption issues aside, evidence suggests that most of ingested calcium may be excreted before reaching the targeted osteoporotic tissues.^21^ To overcome this problem, calcium administration with adjuvant therapy is recommended. Moreover, some researchers suggest that ionic calcium supplements such as antiorbital ionic calcium may provide greater benefit to osteoporosis patients than conventional calcium intake.^22^,^23^ Furthermore, since osteoporosis patients have a high prevalence of vitamin D deficiency, vitamin D supplement becomes a necessity, even in patients receiving AOD.^19^

The course of osteoporosis is mostly silent until it is complicated by fractures. Therefore, primary prevention for osteoporosis, which means treating patients who do not present distinct symptoms, can be hard to maintain. However, maintaining secondary prevention after experiencing OF is expected to be easier. It is anticipated that patients hospitalized due to a traumatic OF experience should be convinced of their need to be treated for osteoporosis. Therefore, secondary prevention compliance is expected to be higher. Nevertheless, compliance rates to osteoporosis drugs are low worldwide.^8^,^24^–^32^

Treatment of osteoporosis is important both before and after OF to prevent subsequent OF and to decrease the mortality rate. Unfortunately, many studies have reported low osteoporosis treatment compliance rates. An observational study from the USA of women experiencing their first hip fracture showed that very few of those patients had undergone osteoporosis assessment/treatment within 6 or 12 months of their OF, only 17% and 23%, respectively.^24^ Panneman et al. reported an anti-osteoporosis treatment usage rate of 17.6% before hospitalization for OF, and half of these patients discontinued treatment in the follow-up period.^25^ In another study by Carnevale et al., 20% of the patients who received osteoporosis treatment before OF discontinued their treatment, and of the patients who had not received any treatment before OF only 40% started treatment after OF.^26^ They also found that 52% of the patients discontinued their osteoporosis treatment after 1.4 years, on average. Tarantino et al. conducted a study of 5,167 patients with hip fractures and found that 61% of the cohort received no medication before and after experiencing a fracture; only 4.5% of the cohort used osteoporosis drugs before OF.^27^ In our study, the usage rate of anti-osteoporosis treatment was higher (29%) before OF compared with other studies. Nevertheless, 17.1% of our patients received no anti-osteoporosis medication, before and after OF.

Although secondary prevention of osteoporosis is important to prevent patients from experiencing fractures, previous studies have shown that the rate of patients receiving anti-osteoporosis treatment was dramatically low. For instance, Kim et al. found that 32.6% of 129 patients who were followed for more than 6 months had received anti-osteoporosis treatment after being diagnosed with a hip fracture.^28^ Another study found that only 24.8% of patients received medication after OF.^27^ In a retrospective study of 47,171 women aged ≥50 years with a fragility fracture, Wilk et al. found that post-fracture only 18% of those patients received osteoporosis treatment within 90 days, and 23% within a year.^29^ Klop et al. studied the trends and determinants of AOD prescribing in 30,516 patients after hip fracture in the United Kingdom. They found that AOD usage rate increased from 7% in 2000 to 46% in 2010. They also found that 94% of the patients treated with anti-osteoporosis drugs also received bisphosphonates.^8^ In our study, 48.6% of the patients received anti-osteoporosis treatment after fracture diagnosis, which was compatible with those previous findings.

Klop et al. reported that bisphosphonates were prescribed for 26% of patients, representing 79% of the osteoporosis prescription medications. Calcitonin was the most frequent non-bisphosphonate therapy in their study and used by 4.8% of the patients; half of the patients (50.3%) used dietary supplements such as calcium, vitamin D, and multi-vitamin. They also found that even after experiencing a fragility fracture, 48.5% of patients reported that they had not been told that they had osteoporosis, and only 33% were prescribed medication for osteoporosis.^8^ In a retrospective study with 65,344 patients, 64.3% of patients received no medication and 35.7% received osteoporosis medication, of which 30.9% (*n*=20,200) of total patients received bisphosphonates and 4.8% (*n*=3,111) of total patients received non-bisphosphonates.^31^ In our study, only two patients who started anti-osteoporosis treatment after fracture were treated with strontium ranelate—one received SERM, and the other was treated with a bisphosphonate.

A study by Giusti et al. reported that, among patients who had undergone surgery for hip fracture and who were started on calcium and vitamin D at discharge, only 36.7% of them continued treatment after 6 months.^32^ In our study, 69.3% of patients who started AOD after fracture were started with calcium and vitamin D, but compliance decreased, ranging from 35.2% to 42.1% in the follow-up period.

The importance of secondary prevention in patients with OF is unquestionable. To the best of our knowledge, our study is the first to examine the OF drug usage compliance among patients in Turkey.

This study had several limitations. First of all, this was a retrospective study. While data acquisition can be difficult in retrospective studies due to data loss issues, we successfully obtained patient data regarding their medication usage for a one-year period. Secondly, this was a single-center study, so our findings cannot be generalized to all of the region. Nevertheless, this study was conducted in one of the largest hospitals in Ankara (750 beds), which treats an average of 6,000 patients per day; the hospital is a trauma center in Ankara which potentially serves a population of five million residents. Another limitation relates to our investigation of drug utilization—data were not available regarding the underlying reasons for the decreasing levels of drug utilization rates. In our study, the entire cohort had access to state health insurance; hence, we can assume that economic reasons were not applicable. However, regardless of the cause, our study found that drug use rates in osteoporosis patients over a one-year period gradually decreased. Particularly in light of this being a worldwide trend, we believe that this should be the subject of future studies. The causes of high dropout rates for secondary osteoporosis prevention clearly require further investigation, and new and comprehensive studies in this field are increasingly important.

In conclusion, although this is a retrospective observational study, we have demonstrated that, among OF patients, AOD usage rates are low and drug cessation rates are high. All clinicians treating osteoporosis patients should be aware of this decline in drug usage rates among those particularly vulnerable patients; awareness of this trend should motivate practitioners to seek out the reasons for this decline, with the aim of achieving a significant decline in subsequent fractures. Physicians should provide more detailed counseling to their patients regarding the use of AOD, the duration of use, and the increased risks associated with discontinuing the medication. It is also important to increase the frequency of doctor–patient meetings so that drug use continues for the desired period. In addition, clinicians should review the drugs prescribed for their patients in terms of osteoporosis risk in order to eliminate the causes of osteoporosis, and, if necessary, drugs should be changed or discontinued according to the treatment needs of the patients. Patients should also be evaluated in terms of diseases in the etiology of secondary osteoporosis, and the underlying diseases, if any, should be treated in order to prevent osteoporosis complications.

## Abbreviations

AOD: anti-osteoporotic drugs
ICD-10: International Classification of Diseases
OF: osteoporotic fractures.
